# A giant peripheral ossifying fibroma of the maxilla with extreme difficulty in clinical differentiation from malignancy: a case report and review of the literature

**DOI:** 10.1186/s13256-024-04529-9

**Published:** 2024-05-04

**Authors:** Ryo Takagi, Kosei Mori, Takashi Koike, Sayumi Tsuyuguchi, Kengo Kanai, Yoshihiro Watanabe, Mitsuhiro Okano, Yoshihiro Noguchi, Aya Tanaka, Kinue Kurihara, Kazumichi Sato, Ken Ishizaki, Yuichiro Hayashi, Yorihisa Imanishi

**Affiliations:** 1 Department of Otorhinolaryngology-Head and Neck Surgery, International University of Health and Welfare, School of Medicine, Narita Hospital, Narita, Japan; 2 Department of Oral Rehabilitation and Maxillofacial Surgery, International University of Health and Welfare, School of Medicine, Narita Hospital, Narita, Japan; 3 Department of Pathology, International University of Health and Welfare, School of Medicine, Narita Hospital, Narita, Japan

**Keywords:** Peripheral ossifying fibroma, Reactive proliferative lesion, Ossification, Periodontal ligament, Periosteum, Differential diagnosis, Terminology, Synonyms

## Abstract

**Background:**

Peripheral ossifying fibroma is a nonneoplastic inflammatory hyperplasia that originates in the periodontal ligament or periosteum in response to chronic mechanical irritation. Peripheral ossifying fibroma develops more commonly in young females as a solitary, slow-growing, exophytic nodular mass of the gingiva, no more than 2 cm in diameter. While various synonyms have been used to refer to peripheral ossifying fibroma, very similar names have also been applied to neoplastic diseases that are pathologically distinct from peripheral ossifying fibroma, causing considerable nomenclatural confusion. Herein, we report our experience with an unusual giant peripheral ossifying fibroma with a differential diagnostic challenge in distinguishing it from a malignancy.

**Case presentation:**

A 68-year-old Japanese male was referred to our department with a suspected gingival malignancy presenting with an elastic hard, pedunculated, exophytic mass 60 mm in diameter in the right maxillary gingiva. In addition to computed tomography showing extensive bone destruction in the right maxillary alveolus, positron emission tomography with computed tomography revealed fluorodeoxyglucose hyperaccumulation in the gingival lesion. Although these clinical findings were highly suggestive of malignancy, repeated preoperative biopsies showed no evidence of malignancy. Since even intraoperative frozen histological examination revealed no malignancy, surgical resection was performed in the form of partial maxillectomy for benign disease, followed by thorough curettage of the surrounding granulation tissue and alveolar bone. Histologically, the excised mass consisted primarily of a fibrous component with sparse proliferation of atypical fibroblast-like cells, partly comprising ossification, leading to a final diagnosis of peripheral ossifying fibroma. No relapse was observed at the 10-month follow-up.

**Conclusions:**

The clinical presentation of giant peripheral ossifying fibromas can make the differential diagnosis from malignancy difficult. Proper diagnosis relies on recognition of the characteristic histopathology and identification of the underlying chronic mechanical stimuli, while successful treatment mandates complete excision of the lesion and optimization of oral hygiene. Complicated terminological issues associated with peripheral ossifying fibroma require appropriate interpretation and sufficient awareness of the disease names to avoid diagnostic confusion and provide optimal management.

## Background

Peripheral ossifying fibroma (POF) is a nonneoplastic inflammatory hyperplasia, that is, a reactive proliferative lesion that arises in the superficial or periapical gingiva, induced by diverse chronic mechanical irritations such as dental calculus, bacterial plaque, orthodontic appliances, ill-fitting crowns and dentures, and improper restorations [1–8]. POF is believed to originate from pluripotent cells of the periodontal ligament or periosteum that can be metaplastically transformed into osteoblasts, cementoblasts, or fibroblasts in response to the aforementioned chronic stimuli [1, 5, 7, 9]. The histopathology is characterized by fibrous connective tissue with varying numbers of fibroblasts associated with the formation of variable amounts of mineralized products consisting of bone components (woven and lamellar bones), cementum-like material, dystrophic calcification, or a combination thereof [1–6, 8, 10]. Although the immunohistochemical profile of POF has been sparsely documented, spindle-shaped cells in POF have been shown to be positive for smooth muscle actin (SMA) in most cases, suggesting a myofibroblastic nature of the lesion [8, 11].

Clinically, POF usually presents as a painless, solitary, slow-growing, relatively well-defined, pedunculated or sessile, exophytic nodular mass of the gingiva [2, 4–9, 12–14]. Epidemiologically, POF develops more commonly in females than in males, mainly during the second to third decades of life, and is predominantly located in the anterior maxilla, especially in the interdental papilla of the incisors [1, 2, 4–8, 13]. Regarding the size, most cases are no more than 2 cm in diameter [2, 5–9, 12–15]; however, very rare cases of POF with unusually marked enlargement (≥ 6 cm) have been reported [11, 16–19], which often require careful differential diagnosis to distinguish them from malignancy.

In clinical practice, there have been nomenclature problems wherein various synonyms have been used to refer to POF, while very similar names also have been applied to neoplastic diseases pathologically distinct from POF, causing considerable confusion among the relevant physicians [2, 4–6, 10, 12, 13, 20].

Here, we report our experience with an unusual giant POF of the maxillary gingiva with a differential diagnostic challenge by reviewing its clinical course and discussing the issues of terminology that should be considered to properly recognize the disease concept of POF.

## Case presentation

A 68-year-old Japanese male presented to our department with an exophytic mass on the right side of the maxillary gingiva that appeared 6 months earlier and had rapidly increased in size. He reported that, although he had upper and lower dentures made by a local dentist approximately 3 years ago, he gave up wearing the upper denture after approximately 6 months because it gradually became ill-fitting. His medical history included high blood pressure and hyperuricemia with orally administered regular medications. He smoked 20 cigarettes per day for more than 35 years and drank 500 mL of beer per day on average for more than 35 years.

**Fig. 1:**
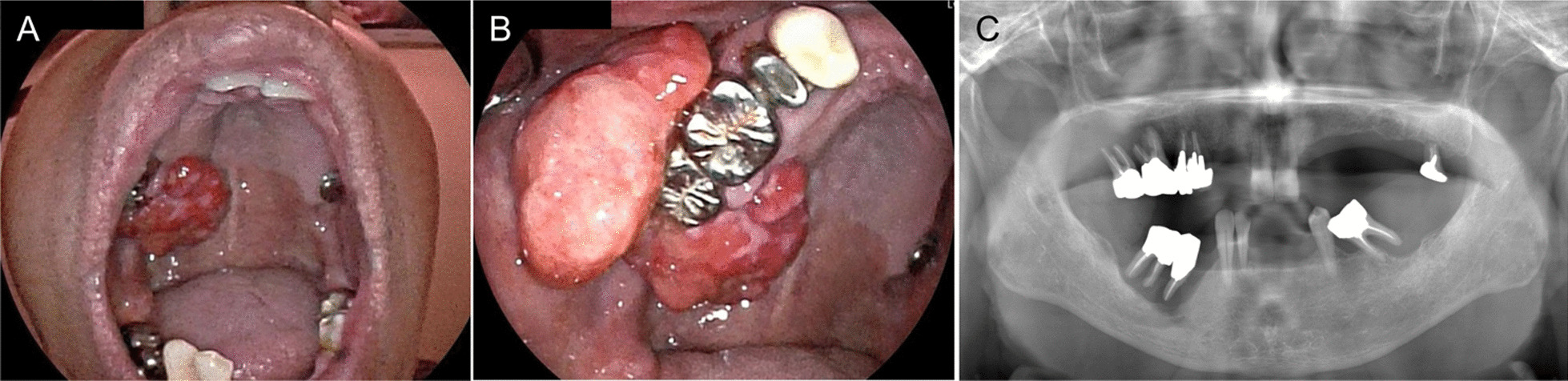
Intraoral and panorama-radiographic findings. **A** and **B** An elastic hard, seemingly well-defined, pedunculated exophytic tumor-like mass with a maximal diameter of approximately 60 mm was observed surrounding the right upper gingiva, including the right upper molars, extending medially from the hard palate, posteriorly to the retromolar trigone, and laterally to the buccal mucosa. **C** Orthopantomogram showing that all the remaining molars and premolars, including those surrounded by the right upper gingival mass, had severe alveolar bone resorption, indicating severe chronic periodontitis

Intraoral inspection revealed an elastic hard, seemingly well-defined, nonhemorrhagic, and almost pedunculated exophytic mass, approximately 60 mm in maximal diameter, extending medially from the hard palate, posteriorly to the retromolar trigone, and laterally to the buccal mucosa, which surrounded the right maxillary gingiva, including the right upper molars (teeth 16 and 17) (Fig. 1A, B). The lesion was painless, and its surface appeared superficially multilobulated and slightly roughened, with some erosions and shallow ulcerations. More than half of the permanent teeth were missing in both the upper and lower jaws, resulting in only five healthy teeth (parts of the maxillary incisors, and the mandibular incisors and cuspids) (Fig. 1C). Cervical palpation found lymphadenopathy of approximately 15 mm in size in the right submandibular region.

**Fig. 2:**
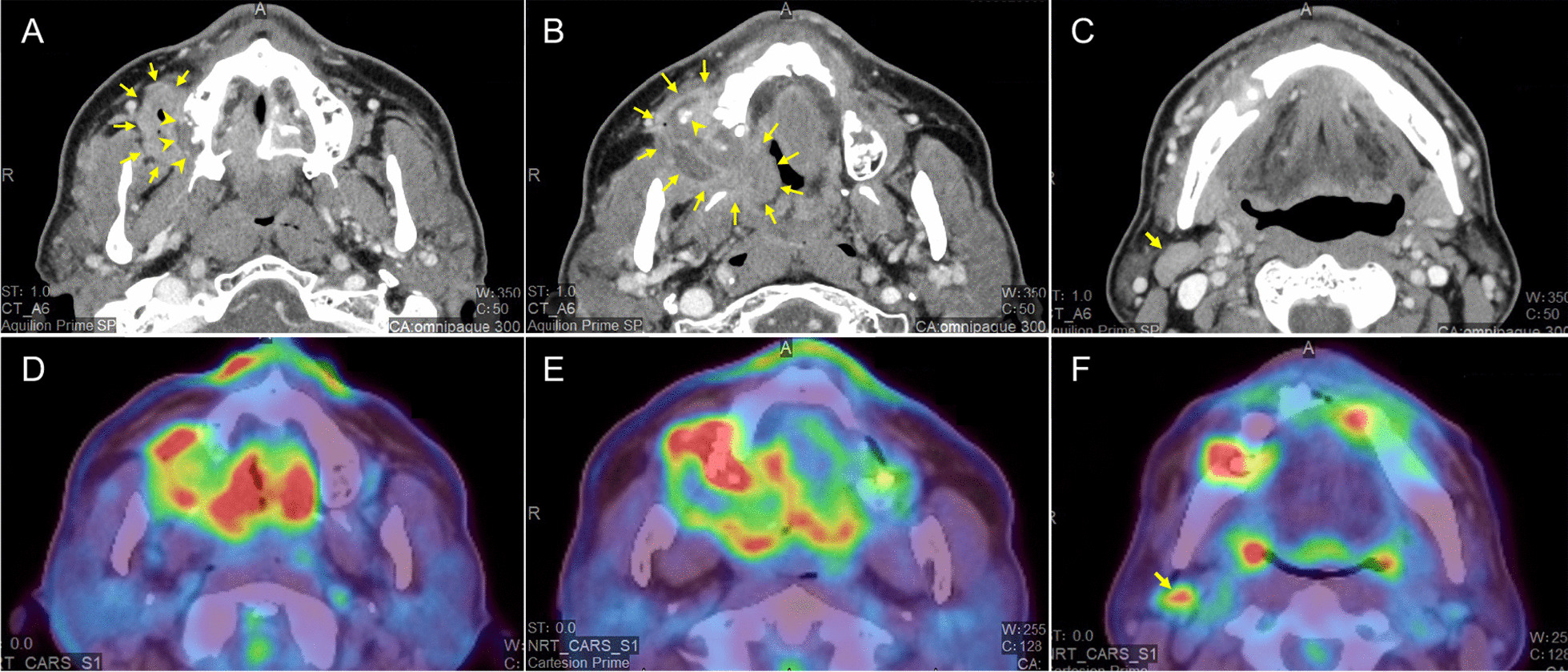
CT and PET/CT findings. **A**–**C** CT image showing a marked bone destruction-like defect on the lateral side of the right maxillary alveolus (arrowhead, **A**) contiguous with the right maxillary gingival mass lesion (arrows, **A** and **B**), along with small calcifications (arrowhead, **B**) anteriorly within the mass. Multiple enlarged lymph nodes, nearly 20 mm in length, were observed in the level I–II region of the right side of the neck (arrows, **C**). **D**–**F** PET/CT scan demonstrating FDG hyperaccumulation (SUVmax = 14.81) in the right maxilla in an area consistent with the gingival lesion containing chronic periodontitis (**D** and **E**). Only mild FDG accumulation was observed in the cervical lymph nodes (arrows, **F**)

An orthopantomogram revealed that, except for the aforementioned healthy teeth, all the remaining molars and premolars, including the molars surrounded by the right upper gingival mass, had severe alveolar bone resorption, indicating that the patient had severe chronic periodontitis (Fig. 1C). Contrast-enhanced computed tomography (CT) revealed extensive bone destruction on the lateral side of the right maxillary alveolus along the medial side of the mass lesion, together with small calcifications anteriorly within the mass (Fig. 2A, B). Multiple enlarged lymph nodes, nearly 20 mm in diameter, were also found in the level I–II region of the right side of the neck (Fig. 2C). Positron emission tomography with CT (PET/CT) revealed noticeable fluorodeoxyglucose (FDG) accumulation (maximum standardized uptake value [SUVmax] 14.81) in the area consistent with the right maxillary gingival mass containing chronic periodontitis (Fig. 2D, E), whereas the right cervical level I–II lymph nodes showed only a relatively mild increase in FDG accumulation (Fig. 2F).

Initial biopsy was performed from the palatal and buccal sides of the surface of the mass, both of which showed “granulation tissue associated with marked inflammatory cell infiltration.” Because the imaging findings suggested a high probability of malignancy, a second biopsy was performed deeper into the lesion; however, the histology showed “severe chronic inflammatory cell infiltration and fibrous connective tissue hyperplasia with some bone tissue involvement,” again with no malignancy. Although pancytokeratin immunostaining was performed, no atypical epithelial cells were observed. At this stage, we additionally considered the possibility of reactive hyperplastic lesions [3, 4] on the gingiva as a differential diagnosis; however, the possibility of malignancy could not be excluded as a pretreatment diagnosis in light of the above-mentioned findings.

**Fig. 3:**
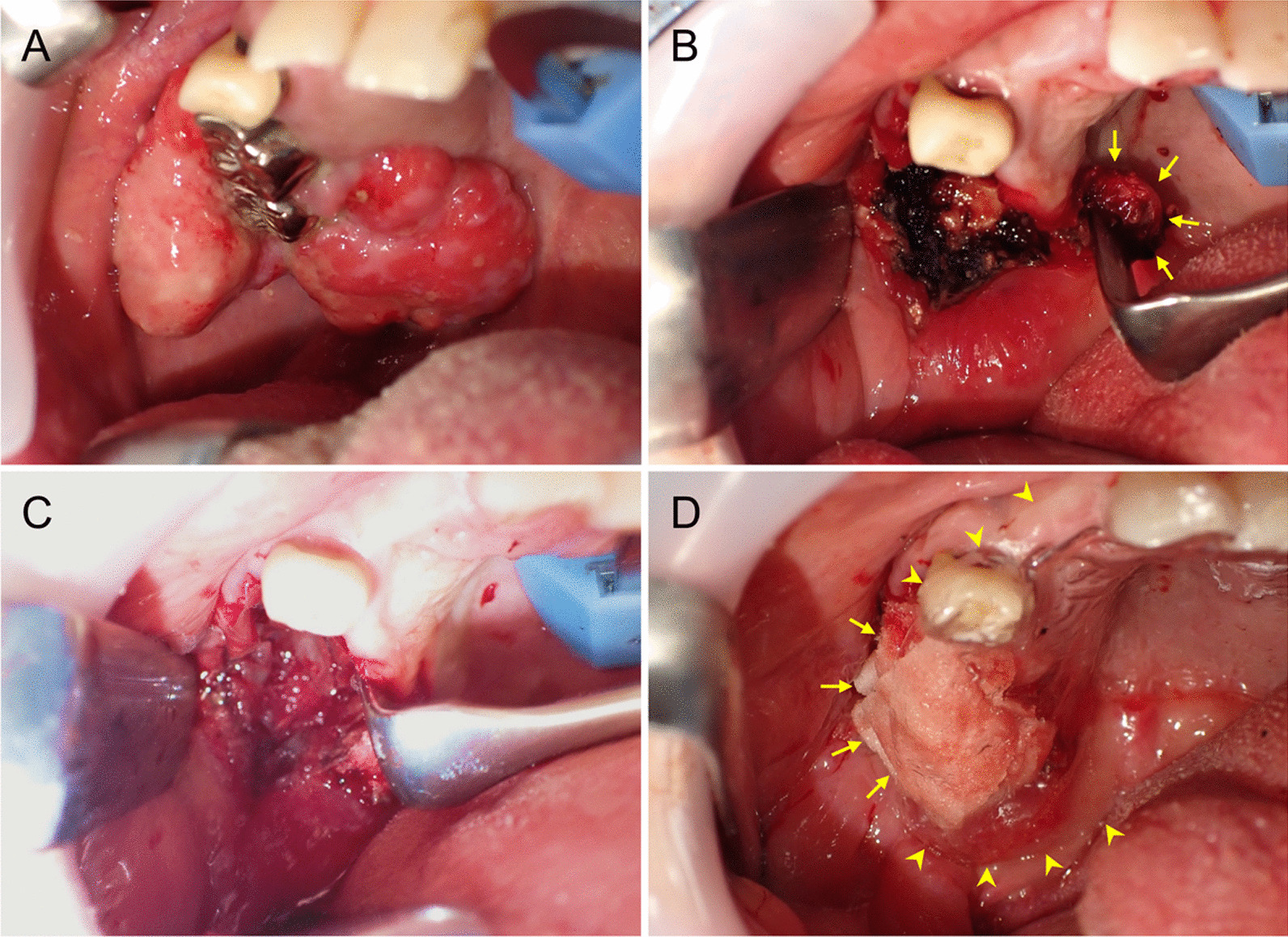
Intraoperative findings. **A** Preoperative appearance of the right maxillary gingival mass lesion. **B** Intraoperative view after resection of the pedunculated gingival mass. The base of the mass was almost confined to the gingival mucosa. The remaining granulation tissues around the resection margin and surrounding alveolar bone were thoroughly curetted (arrow: preserved mucosa elevated from the alveolar bone). **C** The wound surface was covered by a polyglycolic acid sheet with fibrin glue. **D** Following additional covering with a sheet of chitin (poly-*N*-acetylglucosamine)-coated gauze (arrow), an immediate surgical obturator (ISO; arrowhead, transparent in color) was placed

Since surgical resection appeared indispensable regardless of the exact diagnosis, the patient underwent surgery under general anesthesia as a treatment that also served as a definitive diagnosis. Prior to surgery, the aforementioned inactive teeth with severe chronic periodontitis, except for the right maxillary molars contiguous with the lesion, were extracted by a dentist. During surgery, first of all, the two remaining right upper molars and one premolar surrounded by the gingival mass were extracted (Fig. 3A). Then, several small specimens of the mass were excised from the tissue around the extraction socket corresponding to the deepest portion of the lesion and subjected to intraoperative frozen histological examination. Like the preoperative histological findings, all biopsied specimens showed “inflammatory granulation tissue with fibrosis and small calcification” without any malignancy, leading to a provisional diagnosis of ruling out the possibility of malignancy. Accordingly, we decided to perform a procedure similar to partial maxillectomy for benign lesions with minimal resection margins and omitted neck dissection.

**Fig. 4:**
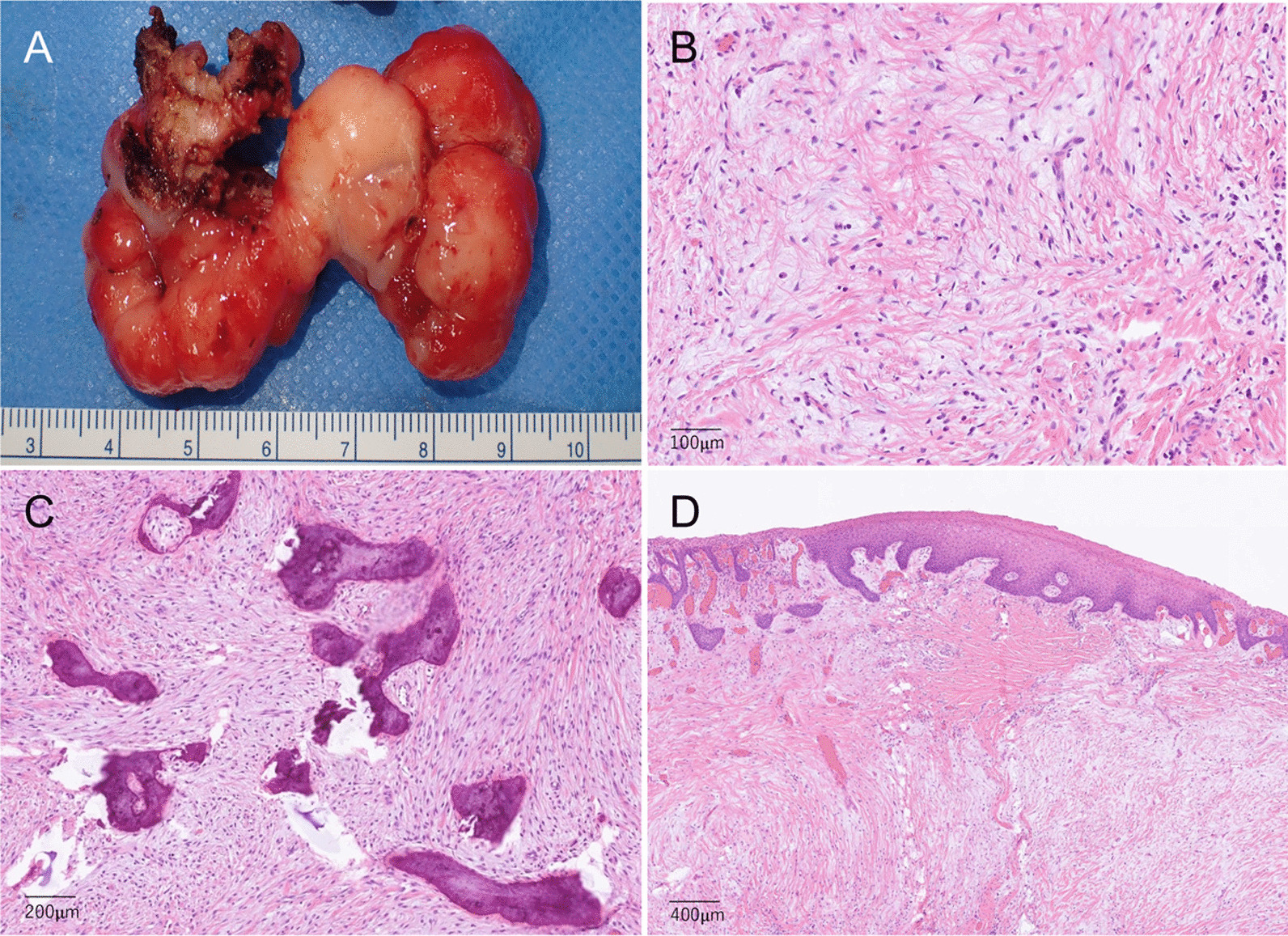
Histopathological findings. **A** The excised mass was partially lobulated and measured approximately 60 × 36 × 17 mm. **B**–**D** Hematoxylin and eosin staining. The histology consisted primarily of a fibrous component with myxoid degeneration and sparse proliferation of atypical fibroblast-like cells (**B**), partly comprising cementum-like ossification and calcification (**C**), without any atypia, even in the superficial squamous epithelium (**D**), leading to a final diagnosis of POF

As resection proceeded, the base of the pedunculated mass was found to be almost confined to the gingival mucosal surface, with the surrounding mucosa remaining normal. After removing the main mass, sufficient detachment and elevation of the surrounding normal mucosa from the periosteum were followed by thorough curettage of the remaining granulation tissues around the resection margin (Fig. 3B). The alveolar bone was sufficiently shaved until a healthy bone margin was exposed, with additional scraping of the sharp edges. Although the bone defect in the maxillary sinus floor extended to approximately 10 mm, the sinus mucosa was preserved without perforation. After meticulous hemostasis, the wound surface was covered by a polyglycolic acid sheet (NEOVEIL Nano^Ⓡ^ D10, Gunze Medical, Japan) with fibrin glue (Fig. 3C) and then with a sheet of chitin (poly-*N*-acetylglucosamine)-coated gauze. An immediate surgical obturator (ISO), premade by the dentist, was placed immediately after surgery (Fig. 3D). The excised mass was partially lobulated and measured approximately 60 × 36 × 17 mm (Fig. 4A).

The histology of the excised mass consisted primarily of a fibrous component with myxoid degeneration and sparse proliferation of atypical fibroblast-like spindle-shaped cells (Fig. 4B), partly comprising cementum-like ossification and calcification (Fig. 4C). No atypia was observed, even in the superficial squamous epithelium (Fig. 4D). Immunostaining revealed mild positivity for SMA in the spindle-shaped cells, whereas S100, desmin, and CD34 were negative. Pancytokeratin staining, for which a positive is suggestive of odontogenic epithelium, was also negative. Based on these histological findings, a final diagnosis of POF was made.

**Fig. 5:**
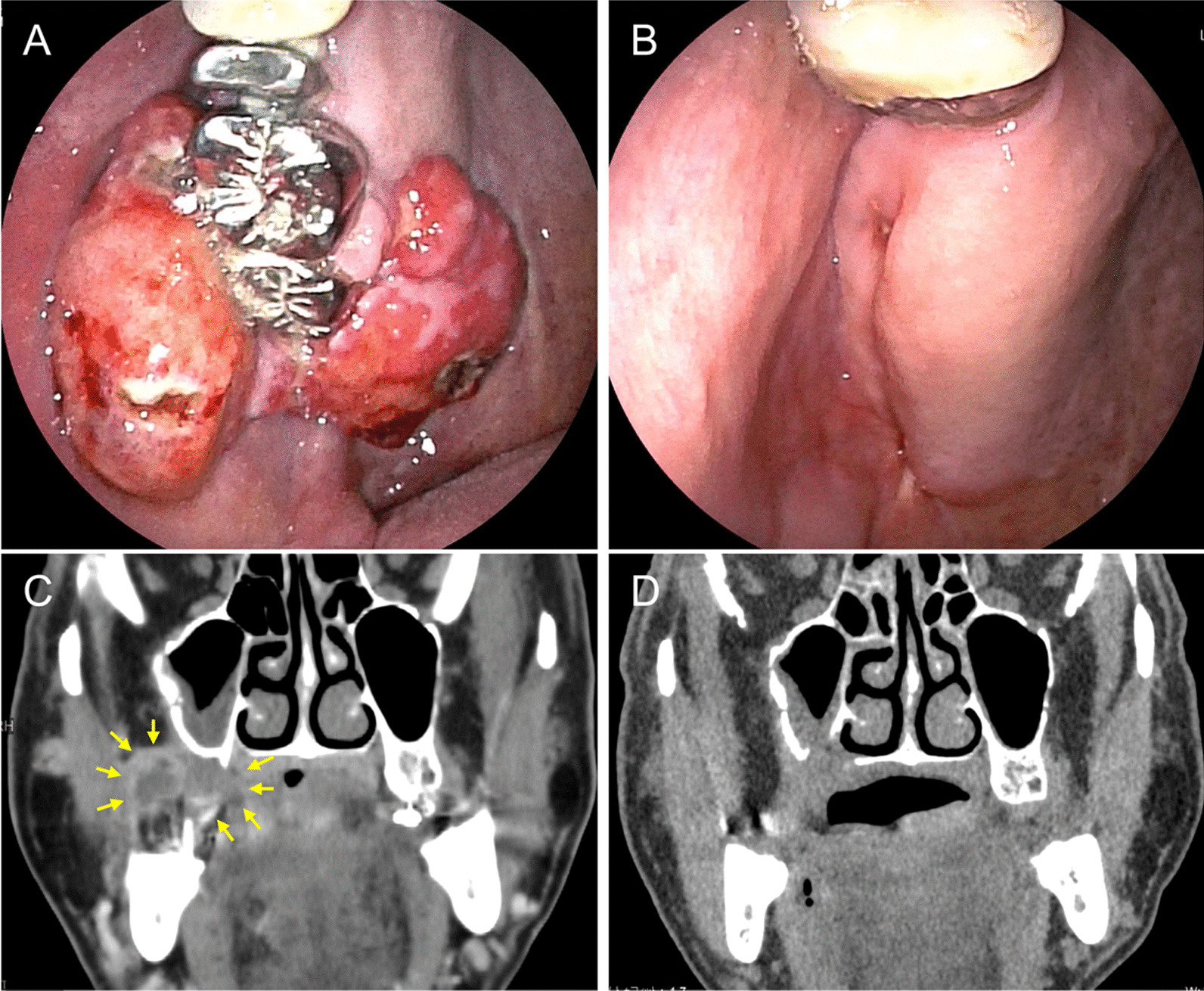
Comparison between pre- and postoperative findings. **A** and **B** Right maxillary gingival lesion site preoperatively (**A**) and 3 months postoperatively (**B**). **C** and **D** Coronal CT images preoperatively (**C** arrow: POF lesion) and 4 months postoperatively (**D** a fistula due to the bone defect of the maxillary sinus floor closed spontaneously)

The surgical wound healed uneventfully with granulation and reepithelialization, thereby maintaining the shape of the alveolar ridge. Three months after surgery, the patient regained the ability to consume a regular diet with the help of dentures remade by the dentist. No relapse or other complications were observed at the 10-month postoperative follow-up (Fig. 5).

## Discussion

We reviewed the POF case series previously reported in various countries and summarized the epidemiological and clinical features (sex, age, site of occurrence, and size) of POF in Table 1 [2, 4, 6–9, 14, 15, 21]. There were sex differences with consistent female dominance, except in one report [7], wherein the female-to-male ratio varied substantially, ranging from 1.3 to 3.5. The second to fourth decades of life were common susceptible ages, with 30s being the average age, and a gradual declining trend in the ratio with aging after 40 years was apparent in large-scale reports [4, 14]. While the occurrence sites were distributed entirely across the upper and lower gingiva, the majority of studies indicated that the anterior maxilla (incisors and cuspids) was the most common site [2, 4, 6, 9, 14, 21]. While the size of lesions ranged quite widely, most studies have reported an average size of 1–2 cm [2, 6, 8, 14, 15] and a maximum diameter of no more than 3 cm [2, 9, 15, 21] or 5 cm [6, 8] (except for a report with unknown data [4]).

**Table 1:** Review of previous articles on POF case series

Ref no.	Year Country	*N*	Sex	Age					Site (%)^a^					Size (cm)
			F (%)	Average (range)	Distribution				Maxilla		Mandible		Unknown	Average (Range)
			M:F		0–9	10–19	20–39	> 40	Ant	Post	Ant	Post		
[2]^b^	2001	134	80 (60)	14	11	123	NA	NA	50 (37)	27 (20)	26 (19)	26 (19)	5 (4)	1.2
	USA		1:1.5	(0–19)										(0.3–3)
[15]	2007	27	16 (59)	38	0	3	12	12	3 (11)	14 (52)	4 (15)	6 (22)	0	1.5
	Taiwan		1:1.5	(15–63)										(0.5–3)
[4]	2010	341	209 (61)	34	3	73	124	98	116 (34)	73 (21)	80 (23)	67 (20)	5 (1.5)	NA
	Israel		1:1.6	(1–90)	(Unknown 43)									
[21]	2012	51	32 (63)	39	0	19	17	15	27 (53)	8 (16)	10 (20)	6 (12)	0	NA
	India		1:1.7	(NA)										(< 1–3)
[6]^c^	2015	27	21 (78)	42	NA	NA	NA	NA	13 (48)		11 (41)		3 (11)	1.3
	Italy		1:3.5	(17–80)					[Ant 16 (59), Post 8 (30)]					(0.3–5.0)
[7]^d^	2016	46	21 (46)	35	NA	NA	NA	NA	19 (39)	12 (25)	4 (8)	14 (29)	0	2.6
	NA		1:0.8	(NA)										NA
[8]^c^	2019	41	23 (56)	38	0	8	16	17	11 (27)		25 (61)		5 (12)	1.2
	Spain		1:1.3	(10–80)					[Ant 13 (32) Post 13 (32), Unknown 15 (37)]					(0.5–5.0)
[9]	2021	23	16 (70)	34	0	4	11	8	10 (43)	2 (9)	2 (9)	5 (22)	0	NA
	Nepal		1:2.3	(10–70)					Both 2 (9)		Both 2 (9)			(0.3–3)
[14]	2022	270	194 (72)	35	7	33	117	96	78 (29)	41 (15)	74 (28)	49 (18)	16 (6)	1.7
	Brazil		1:2.6	(0–87)	(Unknown 12)				Unknown 4 (1.5)		Unknown 8 (3)			(0.2–7)

*F* female, *M* male, *N* number of cases, *NA* not assigned or not available, *Ant* anterior, *Post* posterior

^a^Since the percentages are in principle rounded to the first decimal place, the total sum is not necessarily equal to 100

^b^Report of patients limited to the age of 19 years or younger

^c^Since the numbers of sites were reported only by maxilla/mandible and anterior/posterior, only the total numbers of each are provided

^d^Since some cases had multiple lesions, the sum of the number of cases at each site does not equal the total number of cases

The patient in this report was relatively “elderly” (68 years old) and male, with the lesion located on “the posterior maxilla”; although self-reported, the mass “had grown rapidly to over 6 cm in diameter within 6 months of its initial appearance,” all of which appeared unusual for a POF. In addition, because of the patient’s substantial history of smoking, alcohol consumption, and extremely poor oral hygiene, malignancy was strongly suspected. After treatment, when asked about the history of denture use in detail, the patient told us that, although he had quit using his upper denture due to ill-fitting, he continued to wear only his lower denture for more than 2 years to avoid eating difficulties. Accordingly, inappropriate denture use habits, in which the lower denture provided unnatural chronic mechanical stimulation to the maxillary gingiva during mastication, were suggested to be critical triggering factors for POF development. However, even if we had been aware of this episode from the beginning, there would not have been sufficient evidence to rule out malignancy before treatment.

Regarding the imaging findings of POF, the identification of radiopaque calcified foci via X-ray or CT is likely helpful in differential diagnosis; however, its sensitivity is not sufficient because the amount of calcified tissue varies depending on the patient [5, 7]. Although the preexisting bone structure seldom changes except for compression-associated superficial concave defects and occasional tooth displacement, lesions that have increased in size over time may occasionally present with erosion or even destruction of the bone surface [6, 7, 9]. In the present case, the orthopantomogram showed no radiopaque calcified foci within the lesion, whereas CT displayed a very small number of calcified components in a limited portion of the lesion. However, its small size was not highly indicative of POF, even in hindsight. The marked bone destruction of the maxillary alveolus adjacent to the lesion shown on CT, together with the hyperaccumulation of FDG revealed on PET/CT, appeared to be rather more suggestive of malignancy. In contrast, the findings of preoperative tissue biopsies were, as it turns out, all consistent with POF. Considering that small bone fragments (cementum-like ossification) were contained within the lesion in the second biopsy obtained from a deeper location, it might have been possible to provisionally rule out malignancy at this stage, depending on the degree of experience. However, because of the many unusual features of POF, in terms of its size, clinical course, epidemiological background, and imaging findings suggestive of malignancy, it seemed practically difficult to exclude the possibility of malignancy on the basis of the preoperative examination alone.

In a review of reports of giant cases of POF (consisting of ten cases measuring 2.5 cm or larger) [22], although most required discrimination from malignancy, the proportion of cases with local bone resorption and that of cases with tooth displacement within the lesion were both at most half, suggesting that we should recognize the difficulty of pretreatment differential diagnosis in such giant POFs, as experienced in the present case. Regarding the differential diagnosis from other inflammatory proliferative lesions of the gingiva, peripheral giant cell granuloma (PGCG) is most similar to POF in that it is a reactive lesion that originates exclusively in the periodontal ligament or periosteum of the gingiva [4]. PGCG can be distinguished from POF by its common development in females between the fourth and sixth decades of life, its presentation as a relatively soft nodular mass, and its histological features consisting of a proliferation of mesenchymal cells and multinucleated giant cells associated with prominent vascular growth [4, 9, 23]. However, approximately one-third of PGCG also contains bone components [4, 23], indicating that caution is still needed to distinguish them from each other.

Although conservative local resection is the standard treatment for POF, complete excision of the lesion, including the adjacent periodontal ligament or periosteum where the POF originates, as well as removal of the source of the irritating stimuli, are essential to eliminate the chances of recurrence [2, 6, 8, 9, 14]. In the present case, since no malignancy was reported even on intraoperative histological examination, the resection margin was determined to be as minimal as necessary in accordance with benign tumors. However, to eradicate the possible residual lesions, additional shaving and scraping of the alveolar portion of the maxilla were performed beyond the depth at which the healthy bone was exposed.

Through our experience with this case, we undeniably recognized three possible pitfalls associated with the terminology of POF that should be noted when correctly diagnosing POF and better understanding its pathogenesis. First, the disease conventionally referred to as “ossifying fibroma” means a benign tumor of bone origin whose pathogenesis is entirely different from that of POF. The origin of ossifying fibroma is the periodontal ligament (which is in common with POF) or endosteum (a very thin connective tissue layer covering the bone marrow cavity inside the bone cortex), which principally expands into the medullary space of the bone [3, 6, 7, 12]. Since ossifying fibroma is sometimes referred to as “central ossifying fibroma” (COF) when it needs to be clearly distinguished from POF, it should be noted that the terms “central” versus “peripheral” in this context are employed simply in the sense of indicating their positional relationship in the bone structure [13]. Furthermore, the term “ossifying fibroma” can be referred to in multiple senses (in both broad and narrow senses); it is generally used in the narrow sense to refer to COF, whereas it is sometimes used in the broad sense as an umbrella term for both COF and POF, making the interpretation of this term quite confusing and ambiguous, which requires us to carefully distinguish the meaning indicated by the term depending on the situation [6, 13].

Second, a multitude of synonyms have been used in the nomenclature of POF. Those seen in previous papers are as follows: “peripheral cemento-ossifying fibroma,” “ossifying fibro-epithelial polyp,” “peripheral fibroma with osteogenesis,” “peripheral fibroma with cementogenesis,” “peripheral fibroma with calcification,” “calcifying or ossifying fibroma epulis,” “calcifying fibroblastic granuloma,” “ossifying fibrous epulis,” “peripheral cementifying fibroma,” “calcifying fibroma,” “calcified peripheral fibroma,” and “calcified or ossified fibrous granuloma” [2, 5–7, 9, 10, 13, 14]. Most appear to be a combination of terms meaning “ossification” or “calcification,” and “fibroma” or “fibrous.” However, numerous different names used for the identical pathological condition have led to a considerable degree of confusion in clinical practice [4–6, 10, 13], which appears to be the decisive factor in preventing the spread of accurate recognition of POF. Fortunately, in recent years, a consensus has emerged regarding the use of “peripheral ossifying fibroma (POF)” as the English term for this pathological condition, although a few exceptions remain. Furthermore, since the term “fibroma” literally refers to “benign tumor of fibrous connective tissue origin,” nomenclature-wise, the naming of POF (peripheral ossifying fibroma) itself is undoubtedly a misnomer for the inflammatory reactive proliferative lesion. However, revising its designation at this stage seems rather unwise, as it would have a much greater disadvantage of causing additional unnecessary confusion.

Third, POF should also be distinguished from “peripheral odontogenic fibroma,” a different disease for which the same abbreviation “POF” has been applied [12, 20]. Odontogenic fibroma is classified as one of benign mesenchymal odontogenic tumors in the World Health Organization (WHO) classification, which is further divided into endosteal “central odontogenic fibroma” and extraosseous “peripheral odontogenic fibroma” according to their position in the bone structure; both of these conditions are thus entirely different from POF [2, 13]. The distinction between peripheral ossifying fibroma, an inflammatory reactive proliferative lesion, and peripheral odontogenic fibroma, a benign tumor, is quite misleading because they share the same abbreviation, “POF,” which requires caution to not confuse them.

## Conclusions

Although POF is an inflammatory reactive proliferative lesion, its extreme enlargement can cause alveolar bone destruction and hyperaccumulation of FDG on PET/CT, making the differential diagnosis from gingival malignancy difficult. Proper diagnosis relies on the recognition of its characteristic histopathological findings and identification of possible underlying chronic mechanical stimuli, while successful treatment mandates complete resection of the lesion and improvement of problematic oral hygiene. Due to the numerous synonyms for POF and coexistence of very similar names for different neoplastic diseases, appropriate interpretation and sufficient awareness of these disease names are required to avoid diagnostic confusion and provide optimal management.

## Data Availability

The collected data and materials that can identify the patient are not publicly available because of the adequate protection of patient privacy. All other data collected and analyzed during this case study are included in this published article.

## Abbreviations

COF: Central ossifying fibroma
CT: Computed tomography
FDG: Fluorodeoxyglucose
ISO: Immediate surgical obturator
PET/CT: Positron emission tomography with computed tomography
PGCG: Peripheral giant cell granuloma
POF: Peripheral ossifying fibroma
SMA: Smooth muscle actin
SUV: Standardized uptake value
